# Bacillus clausii Bacteremia Following Probiotic Use: A Report of Two Cases

**DOI:** 10.7759/cureus.57853

**Published:** 2024-04-08

**Authors:** David Corredor-Rengifo, Maria E Tello-Cajiao, Fredy A García-Molina, Leonardo F Montero-Riascos, Janier D Segura-Cheng

**Affiliations:** 1 Internal Medicine, Grupo Interinstitucional de Medicina Interna (GIMI1) Universidad Libre, Cali, COL; 2 Internal Medicine, Faculty of Health Sciences, Universidad Nacional, Cali, COL; 3 Infectious Diseases, Clinica Santa Barbara, Palmira, COL; 4 Infectious Diseases, Clinica Imbanaco, Cali, COL

**Keywords:** case report, sepsis, probiotic, daptomycin, bacillus clausii, bacteremia

## Abstract

The use of probiotics to improve bacterial flora and achieve control of diarrheal episodes is a common practice in outpatients and hospitalized patients. In most cases, related adverse events are few and not life-threatening. However, cases of bacteremia associated with the use of these substances have been described, mainly in the pediatric population in which their prescription is more common. Cases of bacteremia and sepsis have also been documented in immunocompetent and immunocompromised adult patients following the use of probiotics. We present the report of two patients who, in the context of diarrhea, received probiotics with *Bacillus clausii* spores during their stay in the intensive care unit. They subsequently developed sepsis and blood-culture-documented bacteremia. Both patients were treated with daptomycin as the final treatment regimen.

## Introduction

Probiotics are widely used in the management of gastrointestinal (GI) pathologies, particularly in entities such as antibiotic-associated diarrhea, diarrhea associated with *Clostridioides difficile* infection, ulcerative colitis in remission, irritable bowel syndrome, and others [1,2]. They have local immunomodulatory and intestinal barrier-protecting effects against the proliferation of harmful pathogens [3,4]. Several species of bacilli have been commercialized for use as probiotics. These include *Bacillus subtilis, Bacillus​​​​​​​ polyfermenticus, Bacillus​​​​​​​ cereus, Bacillus​​​​​​​ coagulans, Bacillus​​​​​​​ pumilus, Bacillus​​​​​​​ licheniformis,* and *Bacillus​​​​​​​ clausii *[5,6]. Despite their potential benefits [7], cases of bacteremia and sepsis associated with the use of these substances have been reported in recent years, even in immunocompetent individuals [8-10]. Adequate treatment of *B. clausii* bacteremia is challenging due to limited therapeutic experience with this pathogen and potential intrinsic resistance to commonly used antibiotics including macrolides, β-lactams, and aminoglycosides [8-12]. In this report, we present the therapeutic experience of two patients who developed bacteremia and sepsis after receiving probiotics to treat diarrhea.

## Case presentation

Case one

A 51-year-old female patient with a history of hypertension and obesity was admitted to the intensive care unit for sepsis due to gastroenteritis. The gastroenteric molecular panel was positive for *Entamoeba histolytica* and admission blood cultures isolated *Streptococcus gallolyticus*. An antibiotic regimen was started with metronidazole 500 mg intravenous (IV) every 8 hours and ceftriaxone 1 g IV every 12 hours. We performed a colonoscopy that reported ulcerative colitis confirmed by biopsies (chronic colitis with ulceration). After 14 days of antibiotic treatment, the patient responded positively, but diarrhea persisted. Therefore, probiotics with *B. clausii* spores (2,000 million/5 mL orally) were administered every 12 hours for two days, plus mesalazine 1 g orally every 8 hours for ulcerative colitis.

**Figure 1:**
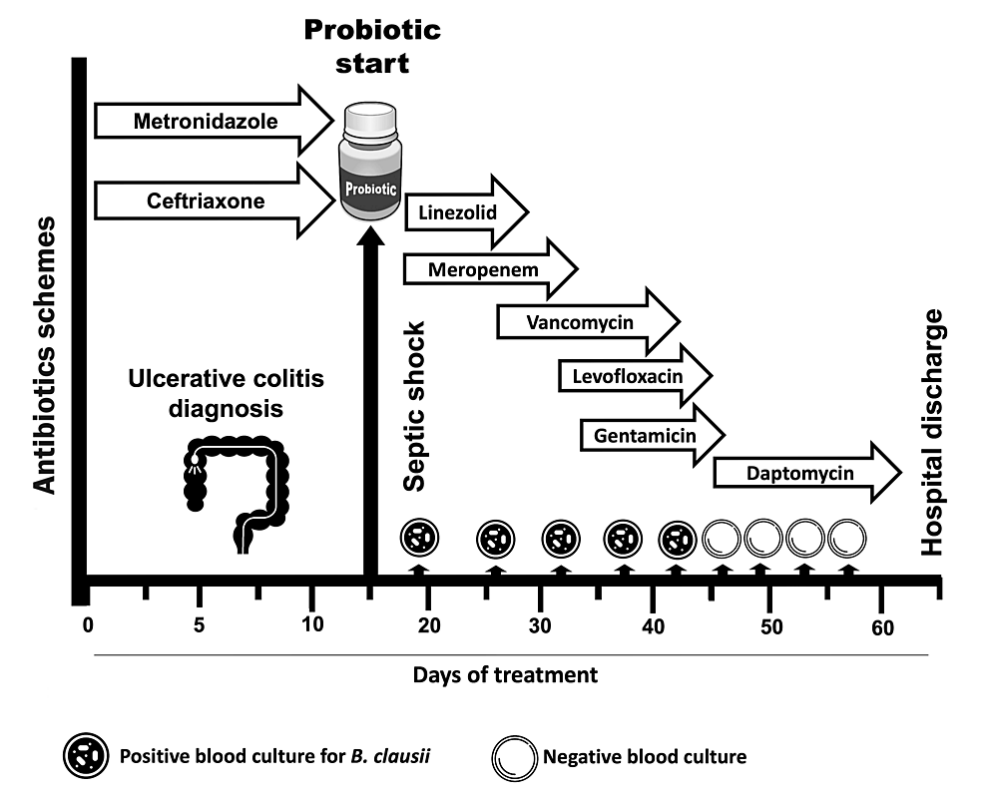
Timeline treatment of case one. The image is created by the authors of this study.

Despite initial clinical improvement, the patient showed rapid deterioration with signs of septic shock requiring ventilatory and vasopressor support. Blood cultures reported Gram-positive bacilli (*Bacillus *spp*.*) typed by matrix-assisted laser desorption ionization-time of flight (MALDI-TOF) ion detector as *B. clausii*. Antibiotics were started with linezolid plus meropenem. With a torpid clinical evolution, several changes in antibiotic regimen had to be made due to the persistence of the systemic inflammatory response and *B. clausii* bacteremia (Figure 1). Infectious foci associated with bacteremia such as endocarditis were also discarded by performing a transthoracic (TT) echocardiogram, in addition to a thoracoabdominal tomography, which ruled out pulmonary or intraabdominal septic embolism, no sign of catheter-related bloodstream infections were documented. After 14 days of multiple antibiotic therapy, we decided to use daptomycin at a dose of 8 mg/kg per day. Negative blood cultures were obtained after 48 hours of treatment. The control blood cultures at 72 and 120 hours of treatment were also negative. The antibiotic was continued for 14 days after the first negative blood culture.

To summarize, the patient was treated with antibiotics for almost seven weeks until microbiological eradication was achieved and then discharged to continue outpatient gastroenterology supervision.

Case two

A 76-year-old female with a history of hypertension, hypothyroidism, and heart failure consulted the emergency department for hypertensive emergency and stroke. During hospitalization, she presented several episodes of diarrhea. Stool culture revealed increased bacterial flora and the presence of polymorphonuclear cells, indicating possible bacterial infection. Ceftriaxone 2 g IV daily for five days was administered, resulting in clinical improvement and subsequent discharge for neurological rehabilitation therapy. Two weeks after her first admission, she returned to the emergency department with diarrhea, abdominal pain, and vomiting. She was admitted to the ICU for septic shock, requiring ventilatory and vasoactive support. Antibiotic treatment was started with meropenem 2 g IV every 8 hours and linezolid 600 mg IV every 12 hours (previous allergy to vancomycin). Molecular tests for gastroenteric pathogens were negative.

**Figure 2:**
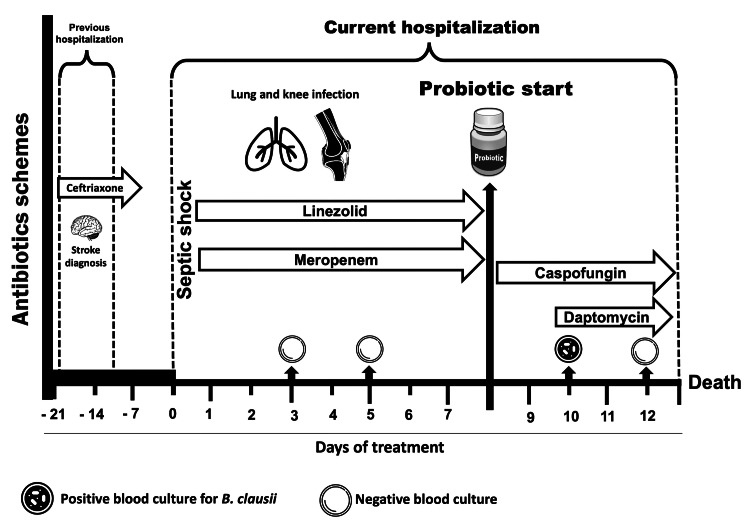
Timeline treatment and interventions in case two. The image is created by the authors of this study.

Two other foci of infection were documented: lobar pneumonia on the right side and a soft tissue infection on the right knee (due to previous trauma during the stroke episode). Surgical lavage of the knee was required, and antibiotics were maintained for eight days. There was no isolation of pathogens in blood cultures or soft tissue samples from the knee. Due to the slow evolution, caspofungin was considered to cover fungal infection. As the patient continued to have episodes of diarrhea, it was decided to start metronidazole and probiotics with *B. clausii* spores (2,000 million/5 mL oral). Without improvement, new blood cultures were obtained. Forty-eight hours after probiotic administration, they were positive for *B. clausii* by MALDI-TOF technique. Administration of daptomycin adjusted to renal function (500 mg every 48 hours) was started, and the probiotic was discontinued. Control blood cultures were negative after 48 hours of treatment, but the patient eventually died (Figure 2).

## Discussion

In this study, we present cases of two patients who developed bacteremia with septic shock after administration of *B. clausii *spore-based probiotics for the treatment of diarrhea. The study by García et al. reported the first Colombian case of bacteremia by this mechanism in an 87-year-old immunocompetent individual [9]. Although reports of bloodstream infections associated with the use of probiotics are rare and mainly described in children, clinicians need to be aware of the possibility of sepsis associated with the use of these agents [8].

In critically ill patients, the potential risk of sepsis with the use of probiotics may be due to transient gastrointestinal dysfunction influenced by several factors, including nutritional status, the use of various medications, such as antibiotics, vasoactive agents, proton pump inhibitors, prolonged hospitalization [13,14], as well as dysfunction of the gastrointestinal system, which may favor the presence of bacterial translocation [15]. This is important because diarrhea is a common condition and there are currently no established protocols for its treatment in critically ill individuals [1,2]. In patients with a diagnosis of ulcerative colitis, as in case one, probiotics could improve the barrier function of the intestinal mucosa, promote the secretion of anti-inflammatory factors, and inhibit the growth of harmful bacteria in the intestine; however, these postulates may not apply to critically ill patients such as those presented in this report [16,17]. Their use is not adequately supported by evidence; therefore, the prescription of probiotics in this setting is an individual decision [7].

Most cases of bacteremia associated with probiotic use report lactobacilli. Therefore, experience with *B. clausii *is even more limited [8]. *B. clausii *is a Gram-positive germ that can be identified by MALDI-TOF. This technique is well suited for the identification of Bacillus species, including those contained in probiotics as spores [18]. The use of antibiotics against Gram-positive bacteria, such as linezolid or vancomycin, is reasonable in the first instance due to the characteristics of the germ [19]. However, because of the persistence of bacteremia and the poor clinical progress, other antibiotics had to be considered.

In case one, a variety of antibiotics including glycopeptides, aminoglycosides, and quinolones were used without achieving negative blood cultures. This reflects the lack of standardization of antibiotic susceptibility and the limited clinical experience in the management of *B. clausii* infections. There is great variability in the reporting of bacterial susceptibility using different regimens, including some antibiotics that are not available in Colombia, such as teicoplanin [9,10]. In addition, the bacterium can develop in vitro resistance to macrolides, beta-lactams, and aminoglycosides [11]. All these factors make it difficult to decide which is the most appropriate scheme to use once *B. clausii* has been typed. Nevertheless, a positive response to antibiotics to which the bacterium is phenotypically resistant has been reported in some cases [8]. In this respect, more research is needed on the ideal treatment scheme for this type of infection.

Based on the persistence of bacteremia in case one and the allergy to vancomycin in case two, we decided to treat the infection with daptomycin, an antibiotic with bactericidal activity against Gram-positive bacteria by a unique mechanism of action. In the presence of calcium, daptomycin alters the cell membrane potential of the bacterium, rendering it unable to generate energy; this allows it to have less cross-resistance with other antibiotics [20]. Both patients achieved negative blood cultures with this antibiotic regimen. In case one, the clinical course was favorable, and the patient was released from the hospital. In case two, the outcome was fatal, possibly related to a lower functional reserve, a greater number of comorbidities, and additional infectious foci. This experience cautions clinicians about the possibility of bacteremia and life-threatening shock with the use of probiotics in severely ill patients.

## Conclusions

The efficacy and safety of using probiotics to treat diarrhea in critically ill patients is unclear. There may be greater gastrointestinal barrier dysfunction in the severe disease state. Thus, the probiotic may become an additional infectious factor. Further research is needed to determine how to use probiotics safely in the critically ill. Since there is no clear treatment against *B. clausii*, there is a variety in the use of treatment regimens, with response time also varying. Daptomycin could be a suitable alternative antibiotic in the first line and those who do not respond to other regimens.
